# Content validation of a new measure of patient-reported barriers to antiretroviral therapy adherence, the I-Score: results from a Delphi study

**DOI:** 10.1186/s41687-022-00435-0

**Published:** 2022-03-26

**Authors:** Kim Engler, Serge Vicente, Kedar K. V. Mate, David Lessard, Sara Ahmed, Bertrand Lebouché

**Affiliations:** 1 Center for Outcomes Research and Evaluation, Research Institute of the McGill University Health Centre, 5252 de Maisonneuve Blvd, Montreal, QC H4A 3S5 Canada; 2 Department of Mathematics and Statistics, University of Montreal, Montreal, QC Canada; 3 School of Physical and Occupational Therapy, McGill University, Montreal, QC Canada; 4 Centre de Recherche Interdisciplinaire en Réadaptation (CRIR), Constance Lethbridge Rehabilitation Center, Montreal, QC Canada; 5 Department of Family Medicine, McGill University, Montreal, QC Canada; 6 Department of Medicine, Division of Infectious Diseases and Chronic Viral Illness Service, McGill University Health Centre, Montreal, QC Canada

**Keywords:** HIV, Patient-reported outcome measures, Adherence, Antiretroviral therapy, Validation, Delphi, Canada, France

## Abstract

**Background:**

Over a third of people living with HIV (PLHIV) have suboptimal adherence to antiretroviral therapy (ART). Measures of barriers to ART adherence often lack comprehensiveness. To help manage ART adherence barriers in HIV care, we are developing a new patient-reported outcome measure (PROM) of these barriers (the I-Score).

**Methods:**

We assessed the content validity of 100 items (distinct barriers) to retain only those most relevant to both PLHIV and HIV health/social service providers. A web-based Delphi was conducted in Canada and France, collecting data from December 2018 to October 2019. Items were evaluated on relevance (the combined rated importance and actionability for HIV care of items among both PLHIV and providers); comprehensibility (rated item clarity); comprehensiveness (examined against our conceptual framework); cross-cultural equivalence (based on comparisons by questionnaire language (English, French) and country of residence). Pearson’s chi-square tests were used for comparisons by language, country, gender, and stakeholder group (PLHIV, providers).

**Results:**

Panelists included 40 PLHIV and 57 providers (66% response rate). Thirty-one items were retained based on consensus thresholds for relevance (minimum: 50% for PLHIV, 60% for providers) and showed good comprehensibility and comprehensiveness, when compared to our conceptual framework (representation of: 6/6 domains, 15/20 subdomains). No significant difference in relevance based on language or country was found among retained items, suggestive of cross-cultural equivalence. Among all 100 items, only 6 significant differences on relevance were observed for gender. For 62 items, the relevance ratings of PLHIV and providers differed significantly, with providers showing greater endorsement of all items but one.

**Discussion:**

The Delphi led to a much-needed item reduction. Remaining items highlight the panel’s multidimensional priorities for the PROM on ART adherence barriers, with few, if any, differences by language, country, and gender. While the analyses may lack generalizability and power, the sample size is considered adequate for a PROM validation study.

**Conclusion:**

Retained items showed good content validity. The different patterns of item endorsement observed underscore the utility of engaging multiple stakeholder groups in PROM development for use in clinical practice. The greater endorsement of items by providers versus patients merits further investigation, including the implications of such differentials for measure development.

**Supplementary Information:**

The online version contains supplementary material available at 10.1186/s41687-022-00435-0.

## Introduction

In the treatment of chronic conditions, not taking medications as prescribed is a widespread and multifactorial problem [1] that is associated with poorer health outcomes and higher healthcare costs [2, 3]. Patient adherence to long-term medications is potentially vulnerable to a wide range of interacting factors related to the health condition; the prescribed therapy; the healthcare team and system; social and economic issues; as well as the patients themselves [4].

Among people living with HIV (PLHIV), the average rate of non-adherence to antiretroviral therapy (ART) worldwide, is estimated to be 37–38% [5, 6]. Addressing adherence barriers in HIV care, while recommended, can be challenging [7]. Impediments include its possible time-intensive nature [8], poor patient-provider communication [9, 10], and inaccurate assessment of patient adherence to ART [11]. The management of adherence barriers may be further challenged by PLHIV and providers having different priorities for HIV care and for addressing adverse health behaviors [12].

To facilitate the identification and discussion of ART adherence barriers that are significant to both PLHIV and providers, our research team is developing the Interference (I)-Score, a new patient-reported outcome measure (PROM) for routine use in HIV care. Two reviews by our team established the need for a more comprehensive measure of these barriers [13, 14]. To this end, a patient-centered conceptual framework was derived from a qualitative synthesis [15] to guide item selection and writing. For use in Canada and France, items were drafted in English and subsequently translated to French, using a multistep process, a multidisciplinary workgroup, and professional translation services (FACITtrans) (see Table 1). All steps were documented including relevant decisional processes which were arrived at collaboratively with FACITtrans or by consensus within the workgroup.

**Table 1:** The I-Score translation process

Step	Actors involved
Two forward translations into the target language (i.e., French)	FACITtrans translators who are native speakers from Canada and France
Reconciliation of the two forward translations by native speaking translators	A multidisciplinary workgroup composed of four bilingual (French, English) I-Score investigators: a physician originally from France (BL), an international student/statistician living with HIV (SV), and two Canadians with backgrounds in the social sciences (KE, DL)
Two back-translations of the reconciled version into English by native English speakers who are fluent in the target language	FACITtrans translators
Review and finalization	The multidisciplinary workgroup of I-Score investigators reviewed the back-translations to finalize the translation
Formatting of the test version in Word format	FACITtrans staff
Proofreading by two translators working independently from one another and reconciling of proofreading	*Proofreading:* One proofreader from FACITtrans One from the workgroup (KE), who discussed results with the other group members for feedback *Reconciliation:* FACITtrans

To reduce and evaluate the content validity of the 100 items, a modified Delphi was conducted with a panel of PLHIV and health and social service providers in Canada and France (for full methodological details see [16]). This step as well as our other stakeholder engagement activities [17, 18] recognize that a PROM’s content validity is crucial [19] and, when seeking PROM implementation in care, so is involving both patients and clinicians in their development [20]. Content validity, the extent to which an instrument’s content reflects its targeted construct, is considered the most important measurement property of a PROM [19]. It refers to its perceived relevance, comprehensiveness, and comprehensibility. The purpose of this paper is thus to present the results of the Delphi’s first round of consultations and answer the following research questions:

1. Which items (i.e. barriers) are deemed by the panelists to be both important and actionable for HIV care?
  To what extent:
2. Are these items considered clearly written?
3. Do importance and actionability scores differ by stakeholder group (i.e. are scores consistent across PLHIV and providers as well as gender)?
4. Do the items show cross-cultural equivalence (i.e. are scores consistent across country and language)?

## Methods

The first round of the Delphi was conducted with an online survey. Participants completed the survey in their language of choice (English or French). To characterize the panel, they first provided sociodemographic and other descriptive information. Then, for each of the 100 items, they rated its: clarity (“Is the item clearly written/does it make sense?”), importance as a barrier to ART adherence (“Is this an important barrier to adhering to antiretroviral therapy?”), and relevance for HIV care or actionability (“Is this useful information for HIV care?”), with a four-point response scale (1-no, 2-somewhat, 3-quite, 4-very). The precise instructions provided to participants in the survey can be found in Additional file 1: Fig. S1.

### Consensus

Round 1 served to class items as: (1) “retained” items: consensus items to consider as core items of the measure; (2) “rejected” items; or (3) “undecided” items to be submitted to Round 2 of the Delphi for consideration as part of an item pool or bank, as done in other work on adherence measures [21]. An item was retained if at least 50% of PLHIV and 60% of providers rated it 3 (quite) or 4 (very) on both importance and actionability. Hence, for retention, the item not only had to be a legitimate barrier to each group, but it needed to be perceived as informative for HIV care. Initially consensus was set at 60% for all panelists. However, the threshold for PLHIV was lowered to 50% as too few items would have been retained. With a 60% threshold, PLHIV would have reached consensus on only 9 items versus 69 items among providers. Furthermore, PLHIV did not achieve a consensus level above 70%. In comparison, with a 50% threshold, consensus among PLHIV is attained for 34 items, reducing the dramatic discrepancy with provider ratings, and improving comprehensiveness. As to rejected items, they were those for which neither group attained the threshold of consensus. Undecided items were those in which the threshold was reached in only one group. As the objectives of the first Delphi round are distinct from those of the second round, a manuscript devoted only to Round 1 is justifiable.

### Participants

HIV providers were recruited, with personalized email invitations, from the participating sites of the I-Score Study (or PROM development study) and from the investigators’ networks. Providers from the following categories were sought: physicians, nurses, pharmacists, psychologists/psychiatrists, social workers and community-based organization staff. They needed to have at least 5 years of experience providing services to PLHIV. PLHIV were recruited from the same sites, from included providers, and from community-based organizations. If interested, their emails were forwarded to the research coordinator who emailed them information on the Delphi. This included briefing interested PLHIV on how to complete the survey, to properly orient their responses:

“***Please note***: In the Delphi, you will be asked to rate the questionnaire items on their importance (i.e. is the particular barrier to adhering to HIV medication important?). When doing so, consider the item’s importance to you and to people living with HIV, *in general*. Your role is to evaluate the questionnaire, not your own personal barriers to adhering to HIV medication.”

PLHIV were required to be at least 18 years of age, to have at least 12 months of experience on ART, and to self-report difficulty taking their ART in the past 5 years. A minimum of 25% women was established for PLHIV and providers. At least 50% of each group recruited in Canada was to be anglophone. Finally, a minimum of three metropolitan cities needed to be represented within each country.

### The survey

The survey was designed with SurveyMonkey Inc. Email invitations were sent to eligible individuals which contained a survey link. A reminder was sent if the survey was not initiated after one week of sending the email. Prior to beginning the survey, participants indicated their informed consent. Item presentation was ordered and contextualized based on the domains and subdomains of the measure’s conceptual framework. After rating each item, a free-text space was provided for comments. All Delphi panelists were compensated for their participation.

### Data analysis

The Delphi panelists were described with sociodemographic variables, split into two groups: PLHIV and providers. These variables were summarized with absolute and relative frequencies.

The observed ratings of the Delphi panelists for each of the 100 items were summarized by the proportion of the panelists who rated an item 3 (quite) or 4 (very), on both importance and actionability. In line with the study objectives, the proportions were split into several pairs for comparison: PLHIV versus providers, females versus males, English versus French questionnaire, and Canada versus France. The proportions were compared with Pearson’s Chi-square test for 2 × 2 contingency tables [22], to assess the null hypothesis of no difference between two proportions. For items where more than 20% of the cells of the corresponding contingency table had expected frequencies less than 5, a Fisher’s exact test was used [23]. The *p*-values of the tests are reported for each item, with a significance level of 5%, acknowledging that this may encompass borderline results [24].

Respondent comments were organized by item and by group (PLHIV versus providers) in table form to detect and describe patterns. They are reported, as needed, to help contextualize the quantitative findings.

## Results

### Response rate

Participants were recruited from December 2018 to October 2019. Of 147 invitations sent, 120 individuals opened the email, while 106 began the survey, and 97 completed it, for a total response rate of 66%. In all, 57 providers and 40 PLHIV formed the panel. It took panelists, on average, 1 h and 44 min to complete the survey.

### Panel description

The mean age of PLHIV was 45.1 (SD: 16.1, Range: 20–72) while the mean age of providers was 44.3 (SD: 10.3, Range: 30–68). Additional characteristics of participating PLHIV and providers are presented in Table 2 which also shows that our recruitment goals for gender and language were met. Overall, the panel’s composition included more respondents who completed the questionnaire in French (55%, English: 45%), Canadian residents (58%, France: 42%), and females (62%, males: 36%, trans: 2%).

**Table 2:** Sociodemographic characteristics of the Delphi panelists (n = 97)

Characteristic	People with HIV	Service providers
	n/40 (%)	n/57 (%)
*Country of residence*		
Canada	24 (60)	33 (58)
France	16 (40)	24 (42)
*Gender*		
Female	22 (55)	38 (67)
Male	16 (40)	19 (33)
Trans	2 (5)	0 (0)
*Survey language*		
English	16 (40)	28 (49)
French	24 (60)	29 (51)
*Health and social service provider*		
Nurse	–	14 (24)
Physician		13 (23)
Pharmacist		13 (23)
Social worker		8 (14)
Community organization staff		5 (9)
Psychologist or psychiatrist		4 (7)
*City*		
Montreal (Quebec)	21	24
Toronto (Ontario)	–	5
St-Jérôme (Quebec)	3	3
Vancouver (British Columbia)	–	1
Paris (France)	7	9
Lyon (France)	–	5
Nantes (France)	2	5
Clermont-Ferrand (France)	5	3
Tourcoing (France)	–	1
Missing (France)	2	1
*Education*		
Primary	4 (10)	–
Secondary	9 (22)	
College/CEGEP/Technical degree	13 (33)	
University	11 (28)	
Other	2 (5)	
Missing	1 (2)	
*Sexual orientation*		
Heterosexual	23 (57)	–
Homosexual	13 (33)	
Bisexual	4 (10)	
*History of drug use*		
Yes	10 (25)	–
*Years providing HIV care/services*		
5–9	–	28 (49)
10–14		8 (14)
15–19		8 (14)
≥ 20		12 (21)
Missing		1 (2)
*Location of practice*		
Hospital	–	36 (63)
Private clinic		7 (12)
Both		12 (21)
Missing		2 (4)

### Objective 1: The most important and actionable items

Table 3 provides decisions on the 100 items (i.e. retained, undecided, rejected). Thirty-one items (31%) met the consensus criteria in both PLHIV and providers. These items covered all six domains of the conceptual framework and were distributed across 15 of its 20 subdomains. Two of the four subdomains of the Healthcare services and system domain were not represented by these items (i.e., HIV clinic issues, Pharmacy issues) as were 2 of the 3 subdomains of the Characteristics of ART domain (i.e., Instructions, Physical features). Panelist comments on the component items of these domains provided little explanation for these results, except for item 98 which five providers remarked was unclear or too vague. The last subdomain not represented was Substance use [composed of two items: Using recreational/party drugs (item 16) and Drinking alcohol (item 15)] within the Lifestyle factors domain. In the panelist comments, for item 16, six providers mentioned the need to consider the quantity and/or type of drug taken (e.g., ‘…a weekend of crystal meth will have a much greater impact on adherence than a joint’ translated from French). Likewise, for item 15, eight provider comments indicated that the key issue for them is the quantity of alcohol consumed (e.g., “Drinking too much?”). Hence, this subdomain’s limited perceived relevance may be explained by some providers’ view that it is the degree of substance use, not necessarily the practice per se, which determines to what extent it qualifies as a barrier.

**Table 3:** Item importance and actionability for HIV care for PLHIV (n = 40) and providers (n = 57) and decisions

Item	Conceptual framework Domain *Subdomain*	Item is important and actionable^a^		*p*-value	Decision^b^
		PLHIV (%)	Providers (%)		
**My activities: Lifestyle factors**					
*Demands and organization of daily life*					
1	A change to my daily routine	33	63	**0.01**	Undecided
2	Travelling	50	56	0.55	Undecided
3	The weekend	20	33	0.15	Rejected
4	**Forgetting**	58	88	**0.01**	**Retained**
5	Work or school	33	40	0.43	Rejected
6	Home or family responsibilities	38	54	0.10	Rejected
7	**An irregular or unpredictable schedule**	55	79	0.22	**Retained**
8	Being too busy	40	53	**0.01**	Rejected
9	**Not being at home**	53	63	0.29	**Retained**
10	My medication schedule conflicting with my sleep pattern	43	72	**0.01**	Undecided
11	My medication schedule conflicting with my eating pattern	33	61	**0.01**	Undecided
12	Having other priorities in my life than taking my medication	33	82	**0.01**	Undecided
13	My medication schedule conflicting with my daily activities	35	79	**0.01**	Undecided
14	Having trouble fitting my medication into my daily life	40	86	**0.01**	Undecided
*Substance use*					
15	Drinking alcohol	33	58	**0.01**	Rejected
16	Using recreational/party drugs	40	72	**0.01**	Undecided
**My thoughts and feelings: Cognitive and emotional aspects**					
*Knowledge*					
17	**Not being informed enough about my medication**	55	65	0.32	**Retained**
18	**Not being sure how to take my medication**	50	75	**0.01**	**Retained**
*Motivation*					
19	**Not feeling motivated to take my medication**	65	88	**0.01**	**Retained**
20	Wanting control over when I take my medication	40	32	0.39	Rejected
21	Wanting control over if I take my medication	45	61	0.11	Undecided
*Acceptance of HIV*					
22	Not wanting to think about having HIV	40	77	**0.01**	Undecided
23	**Having trouble accepting that I have HIV**	53	89	**0.01**	**Retained**
*Beliefs*					
24	Worrying about becoming dependent on my medication	40	30	0.30	Rejected
25	Feeling it is not natural for my mind and body to be taking medication	43	65	**0.03**	Undecided
26	Feeling I can catch up with missed doses	35	54	0.06	Rejected
27	Feeling medication is only for when you feel sick	43	81	**0.01**	Undecided
28	Feeling that stopping my medication for a while is only normal	45	74	**0.01**	Undecided
29	Feeling that I must take my medication my way	48	60	0.24	Undecided
30	Doubting my medication's effects on HIV	38	79	**0.01**	Undecided
31	Being reminded about HIV when taking my medication	48	67	0.06	Undecided
32	**Feeling my medication is toxic or harmful**	55	88	**0.01**	**Retained**
33	Feeling like I have no control over my health	50	49	0.93	Undecided
34	Worrying about taking my medication with recreational/ party drugs or alcohol	35	54	0.06	Rejected
35	Having trouble trusting my medication	40	65	**0.02**	Undecided
36	Having trouble trusting the healthcare system	38	65	**0.01**	Undecided
37	Doubting that I need my medication	38	81	**0.01**	Undecided
38	**Struggling to accept that my medication has both good and bad sides**	50	74	**0.02**	**Retained**
39	Thinking that HIV is a death sentence	48	42	0.60	Rejected
40	Worrying about becoming resistant to my medication	45	33	0.24	Rejected
*Affect*					
41	**Feeling sad or depressed**	60	86	**0.01**	**Retained**
42	Being afraid	48	65	0.09	Undecided
43	Being angry	38	60	**0.03**	Undecided
44	Being worried or anxious	45	56	0.28	Rejected
45	Feeling stressed out	50	56	0.55	Undecided
46	Having mixed (ambivalent) feelings	40	51	0.29	Rejected
47	**Feeling discouraged**	55	72	0.09	**Retained**
48	**Being tired of taking my medication every day**	65	95	**0.01**	**Retained**
**My health: Health experience and state**					
*Bodily signals*					
49	Feeling well	38	19	**0.05**	Rejected
50	**Feeling unwell**	50	67	0.10	**Retained**
51	My body telling me I should not take my medication	40	61	**0.04**	Undecided
52	Feeling my body needs a break from my medication	43	72	**0.01**	Undecided
*Medical signs of HIV and general health*					
53	Getting good test results (viral load or CD4 cell count)	33	16	**0.05**	Rejected
54	**Getting discouraging test results (viral load or CD4 cell count)**	58	63	0.57	**Retained**
55	Having no symptoms of HIV	35	46	0.30	Rejected
56	Having symptoms of HIV	45	32	0.18	Rejected
57	**Being too sick or ill**	60	60	0.97	**Retained**
*Comorbidity*					
58	Having medications to take other than those for HIV	40	54	0.16	Rejected
59	**Having another health condition to deal with (for example, depression. diabetes or heart disease)**	70	74	0.69	**Retained**
**My situation: Social and material context**					
*Relations with others*					
60	**Not getting the support I need from others**	55	72	0.09	**Retained**
61	**Feeling isolated or alone**	55	82	**0.01**	**Retained**
62	**Having relationship problems with someone close to me (for example, conflict or loss)**	53	65	0.22	**Retained**
63	Others discouraging me from taking my medication	33	60	**0.01**	Undecided
64	Being with friends or family	33	37	0.66	Rejected
65	Feeling unloved or unneeded	45	65	**0.05**	Undecided
*HIV stigma and privacy concerns*					
66	Not wanting others to notice that I take this medication	48	88	**0.01**	Undecided
67	**Being concerned about stigma or discrimination related to HIV**	60	88	**0.01**	**Retained**
68	**Fearing rejection because of HIV**	58	79	**0.02**	**Retained**
69	Having privacy or confidentiality concerns related to HIV at my clinic	38	67	**0.01**	Undecided
*Challenging material circumstances*					
70	Having financial problems	40	79	**0.01**	Undecided
71	**Not having a stable or suitable place to live**	63	93	**0.01**	**Retained**
72	**Having trouble getting food or the right kind of food**	58	82	**0.01**	**Retained**
**My medication: Characteristics of antiretroviral therapy**					
*Side effects*					
73	**Worrying about the long-term side effects of my medication**	53	63	0.29	**Retained**
74	Anticipating side effects	38	53	0.14	Rejected
75	**Having side effects from my medication**	55	95	**0.01**	**Retained**
76	**Having side effects that interfere with my daily activities**	63	91	**0.01**	**Retained**
77	**Worrying about my medication’s effects on my physical appearance**	55	68	0.18	**Retained**
*Instructions*					
78	My medication's instructions being too hard to follow	35	54	0.06	Rejected
79	Needing to plan when I eat or find water to properly take my medication	30	58	**0.01**	Rejected
80	Having to take my medication at specific times	38	67	**0.01**	Undecided
81	Finding I have too many pills to take for HIV	43	70	**0.01**	Undecided
*Physical features*					
82	Finding the pills too large	38	68	**0.01**	Undecided
83	Having difficulty swallowing my medication	48	70	**0.02**	Undecided
84	Not liking the taste of my medication	25	51	**0.01**	Rejected
85	Having a problem with the form of the medication (pill. liquid. injection)	30	53	**0.03**	Rejected
**My care: Healthcare services and system**					
*Patient-provider relationship*					
86	Having trouble trusting my primary provider	48	74	**0.01**	Undecided
87	**My primary provider having an unsupportive or negative attitude**	53	82	**0.01**	**Retained**
88	**Not being given enough information by my primary provider about my medication or how to take it**	50	84	**0.01**	**Retained**
89	Having difficulty talking enough with my primary provider	48	70	**0.02**	Undecided
90	**Feeling pressured or powerless with my primary provider in decisions about my health**	50	79	**0.01**	**Retained**
*HIV clinic issues*					
91	Having difficulty getting an appointment at my clinic at the right time	38	67	**0.01**	Undecided
92	Feeling the services at my clinic are not adapted enough to my needs	38	53	0.14	Rejected
93	My clinic's opening hours not being convenient	28	65	**0.01**	Undecided
94	Having trouble getting to my clinic	38	63	**0.01**	Undecided
*Pharmacy issues*					
95	The pharmacy being out of my medication	35	67	**0.01**	Undecided
96	The pharmacy’s opening hours not being convenient	20	49	**0.01**	Rejected
97	Not getting the explanations I need from the pharmacist	38	72	**0.01**	Undecided
98	Having other complaints about the pharmacy services	15	23	0.34	Rejected
99	Having trouble getting to the pharmacy	30	51	**0.04**	Rejected
*Drug cost coverage*					
100	**Not having insurance to cover my medication costs or not having enough coverage**	60	88	**0.01**	**Retained**

^a^Based on scores of 3 (quite) or 4 (very) on both importance and actionability. ^b^Retained: at least 50% of PLHIV and 60% of providers rated each item 3 or 4 on importance and actionability; Rejected: fewer than 50% of PLHIV and fewer than 60% of providers rated each item 3 or 4 on both aspects; Undecided: only one group reached the threshold for retention. In bold: items and decisions in bold indicate retained items, while *p*-values in bold indicate statistically significant group differences in an item's importance and actionability between PLHIV and providers

Twenty-eight items (28%) were rejected, as fewer than 50% of PLHIV and 60% of providers deemed them both important and actionable. They covered all framework domains as well as 14 of its 20 subdomains.

For 41 items (41%), panelists were undecided. For most (n = 39/41, 95%), providers had reached consensus but not PLHIV. All domains were represented by these items as were 17 of the 20 subdomains.

### Objective 2: The clarity of retained items

For all 31 retained items, over two-thirds of each group (PLHIV and providers) deemed the item “quite” or “very” clear. The range of proportions was 68–85% for PLHIV and 67–98% for providers. For details see Additional file 2: Table S1. For two retained items, one group achieved a consensus level below 70% (i.e., 67% among providers for item 57 and 68% among PLHIV for item 90). The panelist comments on item 57, “Being too sick or ill”, showed that five providers wanted greater precision (e.g., ‘AIDS? Too sick to take their Rx? Severe acute illness like a gastro[enteritis]?’ Translated from French). Indeed, for 12 of the 31 items (39%), there was at least one provider comment on the need for greater precision (e.g., ‘In what sense?’ Translated from French, ‘Doesn’t say enough about why’, ‘About something in particular?’). For item 90, “Feeling pressured or powerless with my primary provider in decisions about my health”, PLHIV provided two comments, neither of which helped explain the limited clarity of this item. Hence, it is unknown if these PLHIV clarity ratings signify a problem with item length, terminology (e.g., powerless), or some other issue that complicates understanding.

### Objective 3: Stakeholder group differences

Table 3 shows the results of the comparative analyses of the ratings of PLHIV and providers. For 62 items (62%), a significant difference was observed between the proportions of PLHIV and providers who deemed an item both important and actionable. In every case except one, a greater proportion of providers endorsed the item. The exception was item 53, “Getting good tests results (viral load or CD4 cell count)” (PLHIV: 33% versus Providers: 16%, *p* = 0.05), a rejected item. When examining the comments made by PLHIV on the items in the Delphi, over a third (78/214) were unambiguous descriptions of their personal experience of an ART adherence barrier (e.g., ‘When I am not at home, I do not take my medications because I want to be a normal person’, ‘[I] very seldom drink’). PLHIV emphasis on direct experience with a barrier to determine relevance may thus be contributing to this rating differential with providers on importance/actionability.

The comparison by gender yielded six significant differences (for the full results, see Additional file 3: Table S2). In every case, a greater proportion of males versus females considered the item both important and actionable: Item 7-retained (as per Table 3): An irregular or unpredictable schedule (Females: 58% vs. Males: 89%, *p* = 0.01); Item 22-undecided: Not wanting to think about having HIV (Females: 52% vs. Males: 80%, *p* = 0.01); Item 56-rejected: Not having symptoms of HIV (Females: 28% vs. Males: 49%, *p* = 0.05); Item 69-undecided: Having privacy or confidentiality concerns related to HIV at my clinic (Females: 47% vs. Males: 71%, *p* = 0.02); Item 74-rejected: Anticipating side effects (Females: 35% vs. Males: 66%, *p* = 0.01); and Item 90-retained: Feeling pressured or powerless with my primary provider in decisions about my health (Females: 60% vs. Males: 83%, *p* = 0.02).

### Objective 4: Cross-cultural equivalence

Comparing the items by language of questionnaire completion revealed three significant differences, again, based on the proportion of those considering the item both important and actionable. The results are as follows: Item 24-rejected (as per Table 3): Worrying about becoming dependent on my medication (French: 43% vs. English: 23%, *p* = 0.03); Item 43-undecided: Being angry (French: 60% vs. English: 39%, *p* = 0.03); and Item 53-rejected: Getting good test results (viral load or CD4 cell count) (French: 15% vs, English: 32%, *p* = 0.05).

The analyses by country found 7 significant differences, with 6 items endorsed by a greater proportion of Canadian residents. They included Item 14-undecided: Having trouble fitting my medication into my daily life (Canada: 59% vs. France: 78%, *p* = 0.05); Item 26-rejected: Feeling I can catch up with missed doses (Canada: 59% vs. France: 29%, *p* = 0.01); Item 36-undecided: Having trouble trusting the healthcare system (Canada: 64% vs. France: 39%, *p* = 0.01); Item 44-rejected: Being worried or anxious (Canada: 61% vs. France: 39%, *p* = 0.03); Item 55-rejected: Having no symptoms of HIV (Canada: 52% vs. France: 27%, *p* = 0.01); Item 70-undecided: Having financial problems (Canada: 77% vs. France: 44%, *p* = 0.01); and Item 74-rejected: Anticipating side effects (Canada: 59% vs. France: 29%, *p* = 0.01). For the full results of the cross-cultural analysis see Additional file 3: Table S2.

## Discussion

The general aims of this study were to evaluate the content validity and cross-cultural equivalence of items for a patient-reported measure of barriers to ART adherence through a stakeholder consultation in Canada and France employing Delphi techniques. The content validity of a PROM is essential and relates to its relevance, comprehensiveness, and comprehensibility [19]. Overall, less than a third of items (n = 31) met our relevance criteria, based on the importance and actionability ratings of both PLHIV and HIV health and social service providers. As to comprehensibility, these retained items were deemed clear by over two-thirds of each stakeholder group. Finally, judged against our conceptual framework, retained items covered 100% of domains and 75% of subdomains, suggestive of good comprehensiveness.

### Relevance

Differences in perspectives between PLHIV and their providers have been observed in such areas as HIV care [12], antiretroviral therapy [25], and adherence to ART [11]. For close to two-thirds of items in this study, statistically significant differences were observed in the combined importance and actionability ratings of PLHIV and providers. In almost every case, providers deemed the items more important and actionable. These findings may indicate response bias [26]. For instance, social desirability may lead PLHIV to endorse fewer barriers [27]. Alternatively, as suggested by their comments, many PLHIV may have responded to an item based on whether or not they personally experienced it, thus limiting the number of relevant barriers reported. In contrast, provider responses may reflect years of clinical practice, during which exposure to a wider range of barriers and their impacts is more likely. We addressed this differential and potential threat to our measure’s content validity, by lowering the consensus threshold for PLHIV to 50%, verging on the 51% cutoff recommended by some for Delphi investigations [28]. However, how to respond to and explain such differences between important stakeholder groups in PROM development could be further investigated. Indeed, authors of other Delphi work have underscored the complex elements that can contribute to differences in stakeholder decision-making (e.g., past experience, expectations, cultural factors) and argued for greater attention to this process [29, 30].

Few significant differences in item relevance were based on gender and, when observed, invariably a greater proportion of males endorsed the item. The six items concerned covered all six domains of the conceptual framework. Meta-analyses have established that the male gender is associated with a small advantage in adherence to antiretroviral therapy [31, 32]; however, predictors of adherence in males and females can differ [31]. Further investigation of our PROM with a study powered to analyze the influence of gender is needed to verify these findings. An adequate number of transgender persons could also be included in these analyses. Limited recruitment of this population led to their omission from the comparative analyses in this study. Globally, both transgender men and women are disproportionately burdened by HIV [33] and can face unique barriers to HIV care and adherence to ART (e.g., transphobia, hormone use) [34]. Future research could be designed to assess the content validity of the final PROM specifically among transgender persons.

### Comprehensiveness

The comprehensiveness of adherence barrier measures, both HIV-specific and generic, is often limited [13, 35], which motivated our development of a new PROM. HIV-specific ART adherence barrier measures, on average, have covered approximately a third of subdomains of our conceptual framework and the involvement of patients in their development is reported for less than a third [13]. Items related to healthcare tend to be underrepresented in such instruments relative to those of other domains such as patient- and medication-related factors [13, 21]. Indeed, in our previous work, we found over three-quarters of HIV-specific barrier measures reviewed included no item on the healthcare system or services [13]. While physicians have been criticized for treating non-adherence as a patient problem and responsibility [36], there was little evidence of this in our results. Retained items covered patient factors well (i.e., 12 (29%) items distributed among cognitive and emotional aspects as well as lifestyle factors) but also addressed PLHIV’s social and material context (7 items), their health experience (4 items), the characteristics of ART (4 items), and the healthcare services and system (4 items), with particular emphasis on the patient-provider relationship.

Interestingly, no item based on substance use (Items 15 and 16) was retained. One was rejected (Item 15: Drinking alcohol) while the other was undecided (Item 16: Using recreational/party drugs). This is puzzling as active substance use is reported to be a major predictor of poor ART adherence in PLHIV [37]. Furthermore, a meta-analysis found 13% of adults with HIV reported alcohol and/or substance misuse as a barrier to their ART adherence [38]. As the panelist comments suggest, a lack of emphasis on *problematic* alcohol or drug use (e.g., abuse, addiction) for providers and, among PLHIV, limited personal experience of or willingness to admit these barriers, may be at the source of the items’ dismissal. This will be explored during Round 2 of the Delphi.

### Comprehensibility

The comprehensibility of the retained items, based on the clarity ratings, appeared good overall. However, over a third of retained items were the object of a provider comment requesting greater precision. Ambiguity is certainly a concern when selecting items [26]; it can arise when content is too general, while content that is too specific can limit relevance. An aim with the new PROM is to help initiate and guide patient-provider conversations on ART adherence barriers, which are expected, in turn, to provide details on the patients’ experiences. In short, the PROM is not intended to provide all the answers. Hence, most items were not modified due to these comments. Our measure’s content will be further assessed based on cognitive interviews with PLHIV who, as the targeted respondents of the instrument, are best suited to evaluate its comprehensibility [19]. These will also gather patient input on such aspects as the measure’s instructions, response scale, recall period, length, and appropriateness.

### Cross-cultural equivalence

The cross-cultural validity of patient-reported medication adherence measures is rarely assessed [39]. In this study, comparative analyses on the language of questionnaire completion (English, French) and country of respondents (Canada, France) to evaluate the cross-cultural equivalence of the items only identified significant differences in a few rejected or undecided items. Hence, no differences in perceived importance and actionability, as defined, were found among the retained items, providing encouraging evidence of their applicability to and comparability in settings in both Canada and France.

### Limitations

Several limitations are worth mentioning. Firstly, in this content validation study, as mentioned, the adherence barriers of specific populations with HIV (e.g., gender non-conforming individuals) may not be sufficiently accounted for. Further work will need to evaluate this for the instrument’s widespread applicability. Secondly, statistically, the results we obtained through an analysis of proportions may lack generalizability, due to the moderate sample size and purposive sampling. Non-parametric tests for hypothesis testing were adopted to compensate for this but they can over or underestimate the true difference when the amount of variability differs between the groups and, relative to parametric tests, they have less power. Further research with a larger sample and a parametric approach could help resolve these issues and provide more robust results. Nevertheless, based on criteria advanced by COnsensus-based Standards for the selection of health Measurement INstruments (COSMIN) for a PROM content validity study, our sample size is considered adequate [19]. Thirdly, a protocol deviation occurred in this study resulting in differential consensus thresholds between the panel groups. While unconventional, it seemed necessary to preserve the content validity of the instrument. Finally, redundancies may still be present in the 31 retained items (e.g., 4 items address side effects) and a shorter measure may be more appropriate for use in HIV care. Further consideration of these issues and validation of our instrument with stakeholder consultation is thus in order. Cognitive interviews with 12 additional people with HIV will inform next steps as will the Delphi’s second round.

## Conclusions

This Delphi study provided useful information to reduce the number of items for our measure as well as evidence of the retained items’ content validity and cross-cultural equivalence. It shed light on the ART adherence barriers of greatest interest to both PLHIV and HIV providers in Canada and France which proved multidimensional. Different patterns of item endorsement highlighted the interest of engaging multiple stakeholder groups in measure development [29] but also of further studying their methodological implications.

## Supplementary Information

**Additional file 1.** Instructions provided to Delphi panelists in the Round 1 online survey.

**Additional file 2.** Item clarity ratings of PLHIV and providers.

**Additional file 3.** Item importance and actionability for HIV care by gender, questionnaire language, and country.

## Data Availability

The datasets used and/or analyzed during the current study are available from the corresponding author on reasonable request.

## Abbreviations

ART: Antiretroviral therapy
CD4: Cluster of differentiation 4 (CD4 T helper cells are white blood cells)
CIHR: Canadian Institutes of Health Research
COSMIN: COnsensus-based Standards for the selection of health Measurement INstruments
CTN: Canadian HIV Trials Network
HIV: Human immunodeficiency virus
IISP: Investigator-initiated study program
PLHIV: People living with HIV
PROM: Patient-reported outcome measure
REB: Research ethics board
Rx: A medical prescription
SD: Standard deviation

## References

[1] Mathes T, Jaschinski T, Pieper D (2014). Adherence influencing factors - a systematic review of systematic reviews. Archives of Public Health.

[2] Sokol MC, McGuigan KA, Verbrugge RR, Epstein RS (2005). Impact of medication adherence on hospitalization risk and healthcare cost. Med Care.

[3] Cutler RL, Fernandez-Llimos F, Frommer M, Benrimoj C, Garcia-Cardenas V (2018). Economic impact of medication non-adherence by disease groups: a systematic review. BMJ Open.

[4] Sabaté E (2003) Adherence to long-term therapies. Evidence for action. World Health Organization. Geneva. http://www.who.int/chp/knowledge/publications/adherence_introduction.pdf. Accessed 3 May 2021

[5] Bezabhe WM, Chalmers L, Bereznicki LR, Peterson GM (2016). Adherence to antiretroviral therapy and virologic failure: a meta-analysis. Medicine (Baltimore).

[6] Ortego C, Huedo-Medina TB, Llorca J (2011). Adherence to highly active antiretroviral therapy (HAART): a meta-analysis. AIDS Behav.

[7] Panel on Antiretroviral Guidelines for Adults and Adolescents (2019) Guidelines for the Use of Antiretroviral Agents in Adults and Adolescents Living with HIV. US Department of Health and Human Services (DHHS). https://clinicalinfo.hiv.gov/en/guidelines/adult-and-adolescent-arv/whats-new-guidelines. Accessed 3 May 2021

[8] Genberg BL, Lee Y, Rogers WH, Wilson IB (2015). Four types of barriers to adherence of antiretroviral therapy are associated with decreased adherence over time. AIDS Behav.

[9] Barfod TS, Hecht FM, Rubow C, Gerstoft J (2006). Physicians’ communication with patients about adherence to HIV medication in San Francisco and Copenhagen: a qualitative study using Grounded Theory. BMC Health Serv Res.

[10] Laws MB, Beach MC, Lee Y (2013). Provider-patient adherence dialogue in HIV care: results of a multisite study. AIDS Behav.

[11] Fredericksen R, Crane PK, Tufano J (2012). Integrating a web-based, patient-administered assessment into primary care for HIV-infected adults. J AIDS HIV Res.

[12] Fredericksen RJ, Edwards TC, Merlin JS (2015). Patient and provider priorities for self-reported domains of HIV clinical care. AIDS Care.

[13] Engler K, Lessard D, Lebouché B (2017). A review of HIV-specific patient-reported outcome measures. Patient.

[14] Engler K, Toupin I, Vicente S, Ahmed S, Lebouché B (2019). A review of HIV-specific patient-reported measures of perceived barriers to antiretroviral therapy adherence: what themes are they covering?. J Patient Rep Outcomes.

[15] Engler K, Lènàrt A, Lessard D, Toupin I, Lebouché B (2018). Barriers to antiretroviral therapy adherence in developed countries: a qualitative synthesis to develop a conceptual framework for a new patient-reported outcome measure. AIDS Care.

[16] Engler K, Ahmed S, Lessard D, Vicente S, Lebouché B (2019). Assessing the content validity of a new patient-reported measure of barriers to antiretroviral therapy adherence for electronic administration in routine HIV care: Proposal for a web-based Delphi study. JMIR Res Protoc.

[17] Lessard D, Engler K, Toupin I, Routy JP, Lebouché B, I-Score Consulting Team (2019). Evaluation of a project to engage patients in the development of a patient-reported measure for HIV care (the I-Score Study). Health Expect.

[18] Toupin I, Engler K, Lessard D (2018). Developing a patient-reported outcome measure for HIV care on perceived barriers to antiretroviral adherence: assessing the needs of HIV clinicians through qualitative analysis. Qual Life Res.

[19] Terwee CB, Prinsen CAC, Chiarotto A (2018). Cosmin methodology for evaluating the content validity of patient-reported outcome measures: a Delphi study. Qual Life Res.

[20] Foster A, Croot L, Brazier J, Harris J, O'Cathain A (2018). The facilitators and barriers to implementing patient reported outcome measures in organisations delivering health related services: a systematic review of reviews. J Patient Rep Outcomes.

[21] Kwan YH, Oo L, Loh D (2020). Development of an item bank to measure medication adherence: systematic review. JMIR.

[22] Pearson K (1900). X on the criterion that a given system of deviations from the probable in the case of a correlated system of variables is such that it can be reasonably supposed to have arisen from random sampling. Lond Edinb Dublin Philos Mag J Sci.

[23] Fisher RA (1922). On the interpretation of χ^2^ from contingency tables, and the calculation of P. J Roy Stat Soc.

[24] Hackshaw A, Kirkwood A (2011). Interpreting and reporting clinical trials with results of borderline significance. BMJ.

[25] Yelverton V, Ostermann J, Hobbie A, Madut D, Thielman N (2018). A mixed methods approach to understanding antiretroviral treatment preferences: what do patients really want?. AIDS Patient Care STDS.

[26] Streiner DL, Norman GR, Cairney J (2015). Health measurement scales: a practical guide to their development and use.

[27] Krumpal I (2013). Determinants of social desirability bias in sensitive surveys: a literature review. Qual Quant.

[28] McKenna HP (1994). The Delphi technique: a worthwhile approach for nursing?. J Adv Nurs.

[29] Campbell SM, Shield T, Rogers A, Gask L (2004). How do stakeholder groups vary in a Delphi technique about primary mental health care and what factors influence their ratings?. Qual Saf Health Care.

[30] Brookes ST, Macefield RC, Williamson PR (2016). Three nested randomized controlled trials of peer-only or multiple stakeholder group feedback within Delphi surveys during core outcome and information set development. Trials.

[31] Ortego C, Huedo-Medina TB, Santos P (2012). Sex differences in adherence to highly active antiretroviral therapy: a meta-analysis. AIDS Care.

[32] Langebeek N, Gisolf EH, Reiss P (2014). Predictors and correlates of adherence to combination antiretroviral therapy (ART) for chronic HIV infection: a meta-analysis. BMC Med.

[33] Stutterheim SE, van Dijk M, Wang H, Jonas KJ (2021). The worldwide burden of HIV in transgender individuals: an updated systematic review and meta-analysis. PLoS ONE.

[34] Becasen JS, Morris JD, Denard CL, Mullins MM, Kota KK, Higa DH (2022). HIV care outcomes among transgender persons with HIV infection in the United States, 2006–2021. AIDS.

[35] Chan AHY, Cooper V, Lycett H, Horne R (2020). Practical barriers to medication adherence: what do current self- or observer-reported instruments assess?. Front Pharmacol.

[36] Kleinsinger F (2018). The unmet challenge of medication nonadherence. Perm J.

[37] Socias ME, Milloy MJ (2018). Substance use and adherence to antiretroviral therapy: what is known and what is unknown. Curr Infect Dis Rep.

[38] Shubber Z, Mills EJ, Nachega JB (2016). Patient-reported barriers to adherence to antiretroviral therapy: a systematic review and meta-analysis. PLoS Med.

[39] Kwan YH, Weng SD, Loh D (2020). Measurement properties of existing patient-reported outcome measures on medication adherence: systematic review. JMIR.

