# Supplementary material for: Content validation of a new measure of patient-reported barriers to antiretroviral therapy adherence, the I-Score: results from a Delphi study
Source: J Patient Rep Outcomes. 2022 Mar 26;6:28. doi: 10.1186/s41687-022-00435-0 (PMC8960494; doi:10.1186/s41687-022-00435-0)
Supplement: Supplementary file 1 — Additional file 1. Instructions provided to Delphi panelists in the Round 1 online survey. [file 41687_2022_435_MOESM1_ESM.pdf]

The Delphi Study

Thank you for your interest in our Delphi Study!

Our Delphi study will help us select the most important content for the new measure we are developing. A Delphi collects the opinion of experts, in several rounds through questionnaires, to reach agreement on a topic.

Participating in this Delphi study involves 3 steps:

- **Preparation**
- **Round 1**
- **Round 2**

While completing each step, you may stop and return to it later, using your survey link. The usefulness of the Delphi study depends on participants completing <u>all three steps</u>.

The Delphi Study

The three steps to participating

During Preparation, you will first need to consent to participate. Then, you will complete a short survey on your characteristics.

During Round 1, your main task will be to rate each of the 100 questionnaire items on three aspects:

1) Importance -*Is this an important barrier to adhering to antiretroviral therapy?*
2) Relevance for HIV care -*Is this useful information for HIV care?*
3) Clarity -*Is the item clearly written/does it make sense?*

You can start Round 2 only once you have received, by email, the full results of Round 1. You will get these results several weeks after you complete Round 1. Then, you will need to review these results and fill out the Round 2 questionnaire. This questionnaire will be shorter since it will only contain items on which the Delphi participants disagreed at Round 1.

The Delphi Study

Preparation: participant consent

You have been invited to participate in a Delphi study. To do so, you must provide your consent. By
