# Supplementary material for: Content validation of a new measure of patient-reported barriers to antiretroviral therapy adherence, the I-Score: results from a Delphi study
Source: J Patient Rep Outcomes. 2022 Mar 26;6:28. doi: 10.1186/s41687-022-00435-0 (PMC8960494; doi:10.1186/s41687-022-00435-0)
Supplement: Supplementary file 2 — Additional file 2. Item clarity ratings of PLHIV and providers. [file 41687_2022_435_MOESM2_ESM.docx]

Supplemental Table 1. Item clarity ratings of PLHIV and providers.

| **Item** |  | **Item is clear^a^** | | ***p*-value** | **Decision^b^** |
| --- | --- | --- | --- | --- | --- |
|  |  | PLHIV  (n=40) | Providers  (n=57) |  |  |
| 1 | A change to my daily routine | 65% | 75% | 0.26 | Undecided |
| 2 | Travelling | 80% | 86% | 0.44 | Undecided |
| 3 | The weekend | 63% | 58% | 0.65 | Rejected |
| 4 | **Forgetting** | 78% | 77% | 0.97 | **Retained** |
| 5 | Work or school | 80% | 79% | 0.90 | Rejected |
| 6 | Home or family responsibilities | 73% | 70% | 0.80 | Rejected |
| 7 | **An irregular or unpredictable schedule** | 78% | 86% | 0.28 | **Retained** |
| 8 | Being too busy | 70% | 79% | 0.31 | Rejected |
| 9 | **Not being at home** | 83% | 86% | 0.64 | **Retained** |
| 10 | My medication schedule conflicting with my sleep pattern | 63% | 84% | **0.01** | Undecided |
| 11 | My medication schedule conflicting with my eating pattern | 75% | 82% | 0.37 | Undecided |
| 12 | Having other priorities in my life than taking my medication | 70% | 86% | 0.06 | Undecided |
| 13 | My medication schedule conflicting with my daily activities | 68% | 81% | 0.14 | Undecided |
| 14 | Having trouble fitting my medication into my daily life | 75% | 84% | 0.26 | Undecided |
| 15 | Drinking alcohol | 78% | 77% | 0.97 | Rejected |
| 16 | Using recreational/party drugs | 78% | 86% | 0.28 | Undecided |
| 17 | **Not being informed enough about my medication** | 85% | 86% | 0.89 | **Retained** |
| 18 | **Not being sure how to take my medication** | 75% | 89% | 0.06 | **Retained** |
| 19 | **Not feeling motivated to take my medication** | 80% | 88% | 0.30 | **Retained** |
| 20 | Wanting control over when I take my medication | 63% | 61% | 0.91 | Rejected |
| 21 | Wanting control over if I take my medication | 53% | 61% | 0.38 | Undecided |
| 22 | Not wanting to think about having HIV | 73% | 89% | **0.03** | Undecided |
| 23 | **Having trouble accepting that I have HIV** | 78% | 93% | **0.03** | **Retained** |
| 24 | Worrying about becoming dependent on my medication | 78% | 74% | 0.67 | Rejected |
| 25 | Feeling it is not natural for my mind and body to be taking medication | 73% | 79% | 0.46 | Undecided |
| 26 | Feeling I can catch up with missed doses | 65% | 75% | 0.26 | Rejected |
| 27 | Feeling medication is only for when you feel sick | 65% | 84% | **0.03** | Undecided |
| 28 | Feeling that stopping my medication for a while is only normal | 63% | 82% | **0.03** | Undecided |
| 29 | Feeling that I must take my medication my way | 60% | 67% | 0.50 | Undecided |
| 30 | Doubting my medication's effects on HIV | 75% | 89% | 0.06 | Undecided |
| 31 | Being reminded about HIV when taking my medication | 73% | 86% | 0.10 | Undecided |
| 32 | **Feeling my medication is toxic or harmful** | 73% | 95% | **0.01** | **Retained** |
| 33 | Feeling like I have no control over my health | 68% | 74% | 0.51 | Undecided |
| 34 | Worrying about taking my medication with recreational/ party drugs or alcohol | 70% | 77% | 0.43 | Rejected |
| 35 | Having trouble trusting my medication | 73% | 79% | 0.46 | Undecided |
| 36 | Having trouble trusting the healthcare system | 68% | 88% | **0.02** | Undecided |
| 37 | Doubting that I need my medication | 70% | 81% | 0.22 | Undecided |
| 38 | **Struggling to accept that my medication has both good and bad sides** | 78% | 79% | 0.86 | **Retained** |
| 39 | Thinking that HIV is a death sentence | 70% | 79% | 0.31 | Rejected |
| 40 | Worrying about becoming resistant to my medication | 75% | 75% | 0.96 | Rejected |
| 41 | **Feeling sad or depressed** | 73% | 95% | **0.01** | **Retained** |
| 42 | Being afraid | 68% | 63% | 0.66 | Undecided |
| 43 | Being angry | 70% | 68% | 0.87 | Undecided |
| 44 | Being worried or anxious | 68% | 79% | 0.20 | Rejected |
| 45 | Feeling stressed out | 75% | 84% | 0.26 | Undecided |
| 46 | Having mixed (ambivalent) feelings | 60% | 54% | 0.58 | Rejected |
| 47 | **Feeling discouraged** | 78% | 86% | 0.28 | **Retained** |
| 48 | **Being tired of taking my medication every day** | 78% | 95% | **0.02** | **Retained** |
| 49 | Feeling well | 83% | 67% | 0.08 | Rejected |
| 50 | **Feeling unwell** | 78% | 72% | 0.54 | **Retained** |
| 51 | My body telling me I should not take my medication | 68% | 72% | 0.64 | Undecided |
| 52 | Feeling my body needs a break from my medication | 68% | 84% | **0.05** | Undecided |
| 53 | Getting good test results (viral load or CD4 cell count) | 78% | 77% | 0.97 | Rejected |
| 54 | **Getting discouraging test results (viral load or CD4 cell count)** | 73% | 86% | 0.10 | **Retained** |
| 55 | Having no symptoms of HIV | 70% | 82% | 0.15 | Rejected |
| 56 | Having symptoms of HIV | 70% | 81% | 0.22 | Rejected |
| 57 | **Being too sick or ill** | 80% | 67% | 0.15 | **Retained** |
| 58 | Having medications to take other than those for HIV | 75% | 93% | **0.01** | Rejected |
| 59 | **Having another health condition to deal with (for example, depression. diabetes or heart disease)** | 73% | 91% | 0.09 | **Retained** |
| 60 | **Not getting the support I need from others** | 83% | 84% | 0.82 | **Retained** |
| 61 | **Feeling isolated or alone** | 83% | 95% | 0.09 | **Retained** |
| 62 | **Having relationship problems with someone close to me (for example, conflict or loss)** | 83% | 86% | 0.64 | **Retained** |
| 63 | Others discouraging me from taking my medication | 73% | 81% | 0.34 | Undecided |
| 64 | Being with friends or family | 78% | 68% | 0.33 | Rejected |
| 65 | Feeling unloved or unneeded | 75% | 88% | 0.10 | Undecided |
| 66 | Not wanting others to notice that I take this medication | 78% | 98% | **0.01** | Undecided |
| 67 | **Being concerned about stigma or discrimination related to HIV** | 75% | 93% | **0.01** | **Retained** |
| 68 | **Fearing rejection because of HIV** | 83% | 88% | 0.47 | **Retained** |
| 69 | Having privacy or confidentiality concerns related to HIV at my clinic | 73% | 84% | 0.16 | Undecided |
| 70 | Having financial problems | 75% | 91% | **0.03** | Undecided |
| 71 | **Not having a stable or suitable place to live** | 78% | 98% | **0.01** | **Retained** |
| 72 | **Having trouble getting food or the right kind of food** | 80% | 96% | **0.01** | **Retained** |
| 73 | **Worrying about the long-term side effects of my medication** | 85% | 95% | 0.16 | **Retained** |
| 74 | Anticipating side effects | 75% | 77% | 0.80 | Rejected |
| 75 | **Having side effects from my medication** | 80% | 96% | **0.01** | **Retained** |
| 76 | **Having side effects that interfere with my daily activities** | 83% | 98% | **0.01** | **Retained** |
| 77 | **Worrying about my medication’s effects on my physical appearance** | 83% | 88% | 0.47 | **Retained** |
| 78 | My medication's instructions being too hard to follow | 78% | 89% | 0.11 | Rejected |
| 79 | Needing to plan when I eat or find water to properly take my medication | 73% | 95% | **0.01** | Rejected |
| 80 | Having to take my medication at specific times | 78% | 96% | **0.01** | Undecided |
| 81 | Finding I have too many pills to take for HIV | 83% | 93% | 0.19 | Undecided |
| 82 | Finding the pills too large | 80% | 98% | **0.01** | Undecided |
| 83 | Having difficulty swallowing my medication | 78% | 96% | **0.01** | Undecided |
| 84 | Not liking the taste of my medication | 75% | 98% | **0.01** | Rejected |
| 85 | Having a problem with the form of the medication (pill. liquid. injection) | 75% | 93% | **0.01** | Rejected |
| 86 | Having trouble trusting my primary provider | 78% | 93% | **0.03** | Undecided |
| 87 | **My primary provider having an unsupportive or negative attitude** | 80% | 96% | **0.01** | **Retained** |
| 88 | **Not being given enough information by my primary provider about my medication or how to take it** | 78% | 96% | **0.01** | **Retained** |
| 89 | Having difficulty talking enough with my primary provider | 80% | 89% | 0.19 | Undecided |
| 90 | **Feeling pressured or powerless with my primary provider in decisions about my health** | 68% | 86% | **0.03** | **Retained** |
| 91 | Having difficulty getting an appointment at my clinic at the right time | 78% | 91% | 0.06 | Undecided |
| 92 | Feeling the services at my clinic are not adapted enough to my needs | 78% | 77% | 0.97 | Rejected |
| 93 | My clinic's opening hours not being convenient | 78% | 86% | 0.28 | Undecided |
| 94 | Having trouble getting to my clinic | 73% | 89% | **0.03** | Undecided |
| 95 | The pharmacy being out of my medication | 83% | 95% | 0.09 | Undecided |
| 96 | The pharmacy’s opening hours not being convenient | 75% | 96% | **0.01** | Rejected |
| 97 | Not getting the explanations I need from the pharmacist | 78% | 95% | **0.02** | Undecided |
| 98 | Having other complaints about the pharmacy services | 63% | 54% | 0.43 | Rejected |
| 99 | Having trouble getting to the pharmacy | 75% | 86% | 0.17 | Rejected |
| 100 | **Not having insurance to cover my medication costs or not having enough coverage** | 80% | 96% | **0.01** | **Retained** |

^a^ Based on a rating of 3 (quite) or 4 (very). ^b^ Based on the importance and actionability ratings; Items in bold were retained.
