# Supplementary material for: Content validation of a new measure of patient-reported barriers to antiretroviral therapy adherence, the I-Score: results from a Delphi study
Source: J Patient Rep Outcomes. 2022 Mar 26;6:28. doi: 10.1186/s41687-022-00435-0 (PMC8960494; doi:10.1186/s41687-022-00435-0)
Supplement: Supplementary file 3 — Additional file 3. Item importance and actionability for HIV care by gender, questionnaire language, and country. [file 41687_2022_435_MOESM3_ESM.docx]

Supplemental Table 2. Item importance and actionability for HIV care by gender, questionnaire language, and country.

| **Item** | | **Gender^a^** | | ***p*-value** | **Language** | | ***p*-value** | **Country** | | ***p*-value** |
| --- | --- | --- | --- | --- | --- | --- | --- | --- | --- | --- |
|  |  | Female  (n=60) | Male  (n=35) |  | English  (n=44) | French  (n=53) |  | Canada  (n=56) | France  (n=41) |  |
| 1 | A change to my daily routine | 53% | 49% | 0.65 | 48% | 53% | 0.62 | 50% | 51% | 0.91 |
| 2 | Travelling | 52% | 57% | 0.61 | 59% | 49% | 0.32 | 52% | 56% | 0.67 |
| 3 | The weekend | 25% | 34% | 0.33 | 30% | 26% | 0.73 | 29% | 27% | 0.85 |
| 4 | Forgetting | 75% | 80% | 0.58 | 75% | 75% | 0.96 | 71% | 80% | 0.31 |
| 5 | Work or school | 37% | 40% | 0.75 | 34% | 40% | 0.57 | 30% | 46% | 0.11 |
| 6 | Home or family responsibilities | 45% | 51% | 0.54 | 55% | 42% | 0.20 | 41% | 56% | 0.14 |
| 7 | An irregular or unpredictable schedule | 58% | 89% | **0.01** | 70% | 40% | 0.09 | 73% | 63% | 0.31 |
| 8 | Being too busy | 48% | 49% | 0.98 | 57% | 68% | 0.79 | 52% | 41% | 0.30 |
| 9 | Not being at home | 55% | 66% | 0.31 | 59% | 58% | 0.95 | 57% | 61% | 0.70 |
| 10 | My medication schedule conflicting with my sleep pattern | 62% | 57% | 0.66 | 57% | 62% | 0.59 | 63% | 56% | 0.53 |
| 11 | My medication schedule conflicting with my eating pattern | 53% | 43% | 0.32 | 52% | 47% | 0.62 | 50% | 49% | 0.91 |
| 12 | Having other priorities in my life than taking my medication | 68% | 54% | 0.17 | 55% | 68% | 0.18 | 55% | 71% | 0.12 |
| 13 | My medication schedule conflicting with my daily activities | 67% | 51% | 0.14 | 61% | 60% | 0.92 | 55% | 68% | 0.20 |
| 14 | Having trouble fitting my medication into my daily life | 70% | 66% | 0.66 | 64% | 70% | 0.52 | 59% | 78% | **0.05** |
| 15 | Drinking alcohol | 52% | 43% | 0.41 | 43% | 51% | 0.45 | 45% | 51% | 0.52 |
| 16 | Using recreational/party drugs | 65% | 51% | 0.19 | 59% | 58% | 0.95 | 55% | 63% | 0.43 |
| 17 | Not being informed enough about my medication | 57% | 69% | 0.25 | 61% | 60% | 0.92 | 55% | 68% | 0.20 |
| 18 | Not being sure how to take my medication | 63% | 71% | 0.42 | 70% | 60% | 0.30 | 59% | 73% | 0.15 |
| 19 | Not feeling motivated to take my medication | 78% | 80% | 0.85 | 82% | 75% | 0.45 | 77% | 80% | 0.66 |
| 20 | Wanting control over when I take my medication | 35% | 37% | 0.83 | 30% | 40% | 0.30 | 30% | 41% | 0.26 |
| 21 | Wanting control over if I take my medication | 53% | 60% | 0.53 | 59% | 51% | 0.42 | 52% | 59% | 0.51 |
| 22 | Not wanting to think about having HIV | 52% | 80% | **0.01** | 64% | 60% | 0.74 | 63% | 61% | 0.88 |
| 23 | Having trouble accepting that I have HIV | 75% | 77% | 0.81 | 73% | 75% | 0.76 | 71% | 78% | 0.46 |
| 24 | Worrying about becoming dependent on my medication | 33% | 34% | 0.92 | 23% | 43% | **0.03** | 39% | 27% | 0.20 |
| 25 | Feeling it is not natural for my mind and body to be taking medication | 55% | 60% | 0.64 | 55% | 57% | 0.84 | 61% | 49% | 0.24 |
| 26 | Feeling I can catch up with missed doses | 47% | 46% | 0.93 | 45% | 47% | 0.87 | 59% | 29% | **0.01** |
| 27 | Feeling medication is only for when you feel sick | 67% | 66% | 0.92 | 59% | 70% | 0.27 | 59% | 73% | 0.15 |
| 28 | Feeling that stopping my medication for a while is only normal | 60% | 69% | 0.40 | 59% | 64% | 0.61 | 61% | 63% | 0.79 |
| 29 | Feeling that I must take my medication my way | 57% | 54% | 0.82 | 59% | 51% | 0.42 | 55% | 54% | 0.87 |
| 30 | Doubting my medication's effects on HIV | 63% | 63% | 0.96 | 64% | 60% | 0.74 | 59% | 66% | 0.49 |
| 31 | Being reminded about HIV when taking my medication | 55% | 63% | 0.45 | 55% | 62% | 0.44 | 57% | 61% | 0.70 |
| 32 | Feeling my medication is toxic or harmful | 75% | 74% | 0.94 | 70% | 77% | 0.44 | 71% | 78% | 0.46 |
| 33 | Feeling like I have no control over my health | 53% | 46% | 0.47 | 43% | 55% | 0.26 | 54% | 44% | 0.35 |
| 34 | Worrying about taking my medication with recreational/ party drugs or alcohol | 43% | 51% | 0.45 | 48% | 45% | 0.81 | 54% | 37% | 0.10 |
| 35 | Having trouble trusting my medication | 55% | 57% | 0.84 | 57% | 53% | 0.69 | 50% | 61% | 0.28 |
| 36 | Having trouble trusting the healthcare system | 55% | 51% | 0.74 | 50% | 57% | 0.52 | 64% | 39% | **0.01** |
| 37 | Doubting that I need my medication | 67% | 60% | 0.51 | 64% | 62% | 0.89 | 66% | 59% | 0.45 |
| 38 | Struggling to accept that my medication has both good and bad sides | 58% | 74% | 0.12 | 59% | 68% | 0.37 | 68% | 59% | 0.35 |
| 39 | Thinking that HIV is a death sentence | 40% | 49% | 0.42 | 43% | 45% | 0.84 | 45% | 44% | 0.94 |
| 40 | Worrying about becoming resistant to my medication | 32% | 49% | 0.10 | 36% | 40% | 0.74 | 39% | 37% | 0.79 |
| 41 | Feeling sad or depressed | 73% | 77% | 0.68 | 75% | 75% | 0.96 | 80% | 68% | 0.17 |
| 42 | Being afraid | 55% | 63% | 0.45 | 61% | 55% | 0.51 | 63% | 51% | 0.27 |
| 43 | Being angry | 43% | 63% | 0.07 | 39% | 60% | **0.03** | 48% | 54% | 0.60 |
| 44 | Being worried or anxious | 50% | 54% | 0.69 | 52% | 51% | 0.90 | 61% | 39% | **0.03** |
| 45 | Feeling stressed out | 50% | 60% | 0.35 | 52% | 55% | 0.81 | 61% | 44% | 0.10 |
| 46 | Having mixed (ambivalent) feelings | 42% | 57% | 0.15 | 45% | 47% | 0.87 | 48% | 44% | 0.67 |
| 47 | Feeling discouraged | 62% | 71% | 0.34 | 57% | 72% | 0.13 | 68% | 61% | 0.48 |
| 48 | Being tired of taking my medication every day | 82% | 86% | 0.61 | 84% | 81% | 0.70 | 79% | 88% | 0.24 |
| 49 | Feeling well | 23% | 31% | 0.39 | 32% | 23% | 0.31 | 30% | 22% | 0.36 |
| 50 | Feeling unwell | 55% | 69% | 0.19 | 55% | 64% | 0.34 | 61% | 59% | 0.83 |
| 51 | My body telling me I should not take my medication | 53% | 54% | 0.93 | 43% | 60% | 0.09 | 54% | 51% | 0.82 |
| 52 | Feeling my body needs a break from my medication | 65% | 54% | 0.30 | 55% | 64% | 0.34 | 64% | 54% | 0.29 |
| 53 | Getting good test results (viral load or CD4 cell count) | 17% | 31% | 0.09 | 32% | 15% | **0.05** | 27% | 17% | 0.26 |
| 54 | Getting discouraging test results (viral load or CD4 cell count) | 57% | 69% | 0.25 | 66% | 57% | 0.35 | 63% | 59% | 0.69 |
| 55 | Having no symptoms of HIV | 40% | 43% | 0.78 | 41% | 42% | 0.95 | 52% | 27% | **0.01** |
| 56 | Having symptoms of HIV | 28% | 49% | **0.05** | 36% | 38% | 0.89 | 41% | 32% | 0.35 |
| 57 | Being too sick or ill | 58% | 66% | 0.48 | 68% | 53% | 0.12 | 64% | 54% | 0.29 |
| 58 | Having medications to take other than those for HIV | 48% | 51% | 0.77 | 52% | 45% | 0.49 | 46% | 51% | 0.64 |
| 59 | Having another health condition to deal with (for example. depression. diabetes or heart disease) | 68% | 77% | 0.36 | 73% | 72% | 0.91 | 71% | 73% | 0.85 |
| 60 | Not getting the support I need from others | 67% | 63% | 0.71 | 64% | 66% | 0.81 | 66% | 63% | 0.79 |
| 61 | Feeling isolated or alone | 72% | 71% | 0.98 | 66% | 75% | 0.30 | 73% | 68% | 0.60 |
| 62 | Having relationship problems with someone close to me (for example. conflict or loss) | 58% | 60% | 0.87 | 57% | 62% | 0.59 | 64% | 54% | 0.29 |
| 63 | Others discouraging me from taking my medication | 47% | 54% | 0.47 | 45% | 51% | 0.59 | 50% | 46% | 0.72 |
| 64 | Being with friends or family | 30% | 43% | 0.20 | 34% | 36% | 0.86 | 32% | 39% | 0.48 |
| 65 | Feeling unloved or unneeded | 53% | 63% | 0.37 | 59% | 55% | 0.67 | 63% | 49% | 0.18 |
| 66 | Not wanting others to notice that I take this medication | 70% | 77% | 0.45 | 80% | 64% | 0.10 | 70% | 73% | 0.70 |
| 67 | Being concerned about stigma or discrimination related to HIV | 73% | 83% | 0.29 | 82% | 72% | 0.24 | 77% | 76% | 0.89 |
| 68 | Fearing rejection because of HIV | 68% | 77% | 0.36 | 73% | 68% | 0.61 | 70% | 71% | 0.91 |
| 69 | Having privacy or confidentiality concerns related to HIV at my clinic | 47% | 71% | **0.02** | 57% | 53% | 0.69 | 57% | 51% | 0.56 |
| 70 | Having financial problems | 60% | 69% | 0.40 | 61% | 64% | 0.78 | 77% | 44% | **0.01** |
| 71 | Not having a stable or suitable place to live | 82% | 83% | 0.88 | 77% | 83% | 0.48 | 79% | 83% | 0.59 |
| 72 | Having trouble getting food or the right kind of food | 75% | 71% | 0.70 | 68% | 75% | 0.43 | 75% | 68% | 0.47 |
| 73 | Worrying about the long-term side effects of my medication | 55% | 66% | 0.31 | 55% | 62% | 0.44 | 57% | 61% | 0.70 |
| 74 | Anticipating side effects | 35% | 66% | **0.01** | 52% | 42% | 0.29 | 59% | 29% | **0.01** |
| 75 | Having side effects from my medication | 75% | 89% | 0.11 | 82% | 75% | 0.45 | 77% | 80% | 0.66 |
| 76 | Having side effects that interfere with my daily activities | 78% | 86% | 0.38 | 80% | 79% | 0.97 | 77% | 83% | 0.46 |
| 77 | Worrying about my medication’s effects on my physical appearance | 58% | 71% | 0.20 | 61% | 64% | 0.78 | 66% | 59% | 0.45 |
| 78 | My medication's instructions being too hard to follow | 48% | 46% | 0.81 | 43% | 49% | 0.56 | 41% | 54% | 0.22 |
| 79 | Needing to plan when I eat or find water to properly take my medication | 43% | 54% | 0.30 | 52% | 42% | 0.29 | 46% | 46% | 0.99 |
| 80 | Having to take my medication at specific times | 57% | 54% | 0.82 | 57% | 53% | 0.69 | 55% | 54% | 0.87 |
| 81 | Finding I have too many pills to take for HIV | 62% | 57% | 0.66 | 61% | 57% | 0.64 | 61% | 56% | 0.65 |
| 82 | Finding the pills too large | 63% | 46% | 0.09 | 55% | 57% | 0.84 | 52% | 61% | 0.37 |
| 83 | Having difficulty swallowing my medication | 68% | 51% | 0.10 | 61% | 60% | 0.92 | 63% | 59% | 0.69 |
| 84 | Not liking the taste of my medication | 43% | 37% | 0.55 | 43% | 38% | 0.59 | 39% | 41% | 0.83 |
| 85 | Having a problem with the form of the medication (pill. liquid. injection) | 43% | 46% | 0.82 | 39% | 47% | 0.40 | 41% | 46% | 0.60 |
| 86 | Having trouble trusting my primary provider | 60% | 71% | 0.26 | 59% | 66% | 0.48 | 63% | 63% | 0.93 |
| 87 | My primary provider having an unsupportive or negative attitude | 72% | 71% | 0.98 | 66% | 74% | 0.41 | 68% | 73% | 0.57 |
| 88 | Not being given enough information by my primary provider about my medication or how to take it | 72% | 71% | 0.98 | 70% | 70% | 0.95 | 68% | 73% | 0.57 |
| 89 | Having difficulty talking enough with my primary provider | 57% | 71% | 0.15 | 52% | 68% | 0.12 | 59% | 63% | 0.65 |
| 90 | Feeling pressured or powerless with my primary provider in decisions about my health | 60% | 83% | **0.02** | 59% | 74% | 0.13 | 64% | 71% | 0.50 |
| 91 | Having difficulty getting an appointment at my clinic at the right time | 52% | 63% | 0.29 | 45% | 62% | 0.10 | 57% | 51% | 0.56 |
| 92 | Feeling the services at my clinic are not adapted enough to my needs | 45% | 51% | 0.54 | 39% | 53% | 0.16 | 50% | 41% | 0.40 |
| 93 | My clinic's opening hours not being convenient | 47% | 57% | 0.32 | 48% | 51% | 0.75 | 50% | 49% | 0.91 |
| 94 | Having trouble getting to my clinic | 50% | 60% | 0.35 | 48% | 57% | 0.38 | 52% | 54% | 0.86 |
| 95 | The pharmacy being out of my medication | 60% | 46% | 0.18 | 57% | 51% | 0.56 | 55% | 51% | 0.69 |
| 96 | The pharmacy’s opening hours not being convenient | 38% | 37% | 0.91 | 30% | 43% | 0.16 | 36% | 39% | 0.74 |
| 97 | Not getting the explanations I need from the pharmacist | 60% | 57% | 0.78 | 59% | 57% | 0.81 | 55% | 61% | 0.58 |
| 98 | Having other complaints about the pharmacy services | 22% | 17% | 0.59 | 14% | 25% | 0.18 | 21% | 17% | 0.59 |
| 99 | Having trouble getting to the pharmacy | 42% | 46% | 0.70 | 39% | 45% | 0.51 | 41% | 44% | 0.78 |
| 100 | Not having insurance to cover my medication costs or not having enough coverage | 77% | 80% | 0.71 | 77% | 75% | 0.84 | 80% | 71% | 0.27 |

^a^ The two trans participants were not included.
